# Relationships between serum electrolyte concentrations and ileus: A joint clinical and mathematical modeling study

**DOI:** 10.14814/phy2.14735

**Published:** 2021-02-01

**Authors:** James A. Penfold, Cameron I. Wells, Peng Du, Anna Qian, Ryash Vather, Ian P. Bissett, Gregory O'Grady

**Affiliations:** ^1^ Department of Surgery Faculty of Medical and Health Sciences The University of Auckland Auckland New Zealand; ^2^ Department of Surgery Auckland District Health Board Auckland New Zealand; ^3^ Auckland Bioengineering Institute The University of Auckland Auckland New Zealand

**Keywords:** chloride, electrolytes, Ileus, Interstitial cells of Cajal (ICC), motility, smooth muscle

## Abstract

**Aim:**

Prolonged postoperative ileus (PPOI) occurs in around 15% of patients after major abdominal surgery, posing a significant clinical and economic burden. Significant fluid and electrolyte changes may occur peri‐operatively, potentially contributing to PPOI; however, this association has not been clearly elucidated. A joint clinical‐theoretical study was undertaken to evaluate peri‐operative electrolyte concentration trends, their association with ileus, and predicted impact on bioelectrical slow waves in interstitial cells of Cajal (ICC) and smooth muscle cells (SMC).

**Methods:**

Data were prospectively collected from 327 patients undergoing elective colorectal surgery. Analyses were performed to determine associations between peri‐operative electrolyte concentrations and prolonged ileus. Biophysically based ICC and SMC mathematical models were adapted to evaluate the theoretical impacts of extracellular electrolyte concentrations on cellular function.

**Results:**

Postoperative day (POD) 1 calcium and POD 3 chloride, sodium were lower in the PPOI group (*p* < 0.05), and POD3 potassium was higher in the PPOI group (*p* < 0.05). Deficits beyond the reference range in PPOI patients were most notable for sodium (Day 3: 29.5% ileus vs. 18.5% no ileus, *p* = 0.04). Models demonstrated an 8.6% reduction in slow‐wave frequency following the measured reduction in extracellular NaCl on POD5, with associated changes in cellular slow‐wave morphology and amplitude.

**Conclusion:**

Low serum sodium and chloride concentrations are associated with PPOI. Electrolyte abnormalities are unlikely to be a primary mechanism of ileus, but their pronounced effects on cellular electrophysiology predicted by modeling suggest these abnormalities may adversely impact motility recovery. Resolution and correction of electrolyte abnormalities in ileus may be clinically relevant.

## 1 INTRODUCTION

Delayed GI transit after surgery (postoperative ileus; POI) is common, occurring in 15%–25% of patients undergoing major abdominal surgery, and results in distressing symptoms, prolonged length of hospital stay, and a considerable economic burden (Iyer et al., 2009; Madl & Druml, 2003; Peters et al., 2017). The pathophysiology of POI is multifactorial and remains incompletely understood; implicated mechanisms include the activation of inflammatory pathways, autonomic imbalance, opiate analgesia, and electrolyte abnormalities (Bauer & Boeckxstaens, 2004; Bennink et al., 2008; Carroll & Alavi, 2009; Vather et al., 2014).

Serum electrolyte derangements are common postoperatively, potentially impairing GI motility by their effect on ion channels and cellular membrane potentials, as well as intra‐cellular functions and inter‐cellular co‐ordination. These abnormalities have previously been proposed as a possible factor contributing to prolonged POI (PPOI; Vather et al., 2014). However, the relationship between serum electrolyte concentrations and ICC/smooth muscle cell (SMC) function, which contributes directly to GI motility, has not been clearly defined, and the extent that electrolyte abnormalities contribute to ileus is still unclear. In addition, most management guidelines for ileus to date have focused on the monitoring and correction of sodium, and to lesser extent potassium (Lowman, 1971; Mattei & Rombeau, 2006; Vather & Bissett, 2013). However, extracellular chloride concentrations may also be important, given the recently discovered role of the calcium‐activated chloride channel, Anoctamin‐1 (Ano1) in slow‐wave pacemaking, and the role of the chloride equilibrium potential in modulating slow‐wave morphology (Gomez‐Pinilla et al., 2009; Lees‐Green et al., 2014; Singh et al., 2014).

Mathematical modeling offers a flexible way of investigating the effects of imbalance of extracellular electrolyte concentrations on gut functions in silico. A number of studies have proposed predictive models that can be used for this purpose, particularly focusing on the role of thermal imbalances on GI electrophysiology (Altomare et al., 2014; Gizzi et al., 2010). A number of models have been proposed to investigate the formation of more complex functional circuits such as re‐entry in the gut (Bini et al., 2010; Lammers et al., 2012; Tse et al., 2016). Another aspect of GI mathematical modeling is biomechanics (Brandstaeter et al., 2018; Turkmani et al., 2019), where the dynamics of calcium cycling at the cellular levels have been linked to motility as well as thermodynamics (Gizzi et al., 2015, 2016).

This joint clinical‐theoretical study was undertaken to evaluate trends in peri‐operative electrolyte concentrations, and their relationship to PPOI. Predicted effects of the commonest PPOI electrolyte derangements on ICC and SMC function were evaluated using biophysically based mathematical cell models previously developed and validated for in silico investigations of this type (Du et al., 2016; Lees‐Green et al., 2014; Poh, Corrias, et al., 2012). The aim was to address whether physiological changes in electrolytes in PPOI could contribute to the delayed return of GI motility.

## 2 METHODS

Ethical approval was gained from the New Zealand National Ethics Committee and the local institutional review board.

### 2.1 Clinical data

The study population was sourced from a prior prospective study from Auckland City Hospital (Vather, Josephson, Jaung, Kahokehr, et al., 2015; Vather, Josephson, Jaung, Robertson, 2015). Participants aged 18 and over undergoing elective laparoscopic or open colorectal surgery were screened for eligibility. Procedures undertaken included segmental rectal or colonic resection, total colectomy, formation/reversal of ileostomy/colostomy, and abdominoperineal resection. Blood tests were obtained pre‐operatively, on postoperative day (POD) 1, on POD3 if patients were still in the hospital, and at any further timepoints as per clinical discretion. Serum sodium, potassium, and chloride concentrations were routinely measured, with calcium and magnesium also often available. Results beyond POD4 were not analyzed, as those who developed PPOI were randomly allocated to gastrografin or placebo on POD4 as part of a separate study.

The primary clinical outcome was PPOI occurrence, defined according to a previously published consensus definition (Vather & Bissett, 2013). Participants were deemed to have PPOI if they met two or more criteria on/after POD four: (a) nausea/vomiting, (b) inability to tolerate oral solid/semi‐solid diet over past two meals, (c) lack of flatus and stool over last 24 hr, (d) abdominal distension, or (e) radiological evidence of ileus on CT/abdominal plain film over past 24 hr (Vather et al., 2013). Secondary outcomes included length of stay, minor and major complications as defined by the Clavien‐Dindo classification, and peri‐operative intravenous (IV) fluid administration (Dindo et al., 2004).

### 2.2 Data analysis

Statistical analyses of the clinical data were performed using GraphPad Prism 7 for Windows (Version 7.04) and RStudio for Mac (Version 1.0.153). The Shapiro–Wilk test was used to determine parametricity of examined variables. Chi‐squared tests were used to analyze categorical variables. Parametric data were presented as mean ± standard deviation (*SD*), and analyzed using the Student's *t* test. Non‐parametric data were presented as median ± interquartile range (IQR) and analyzed using a Mann–Whitney *U* test. A Pearson correlation plot was constructed to assess the correlation between changes in different electrolytes. Kruskal–Wallis tests were performed to compare changes in electrolytes to secondary outcomes such as length of stay, and Clavien‐Dindo grade.

### 2.3 Mathematical cell model

**FIGURE 1:**
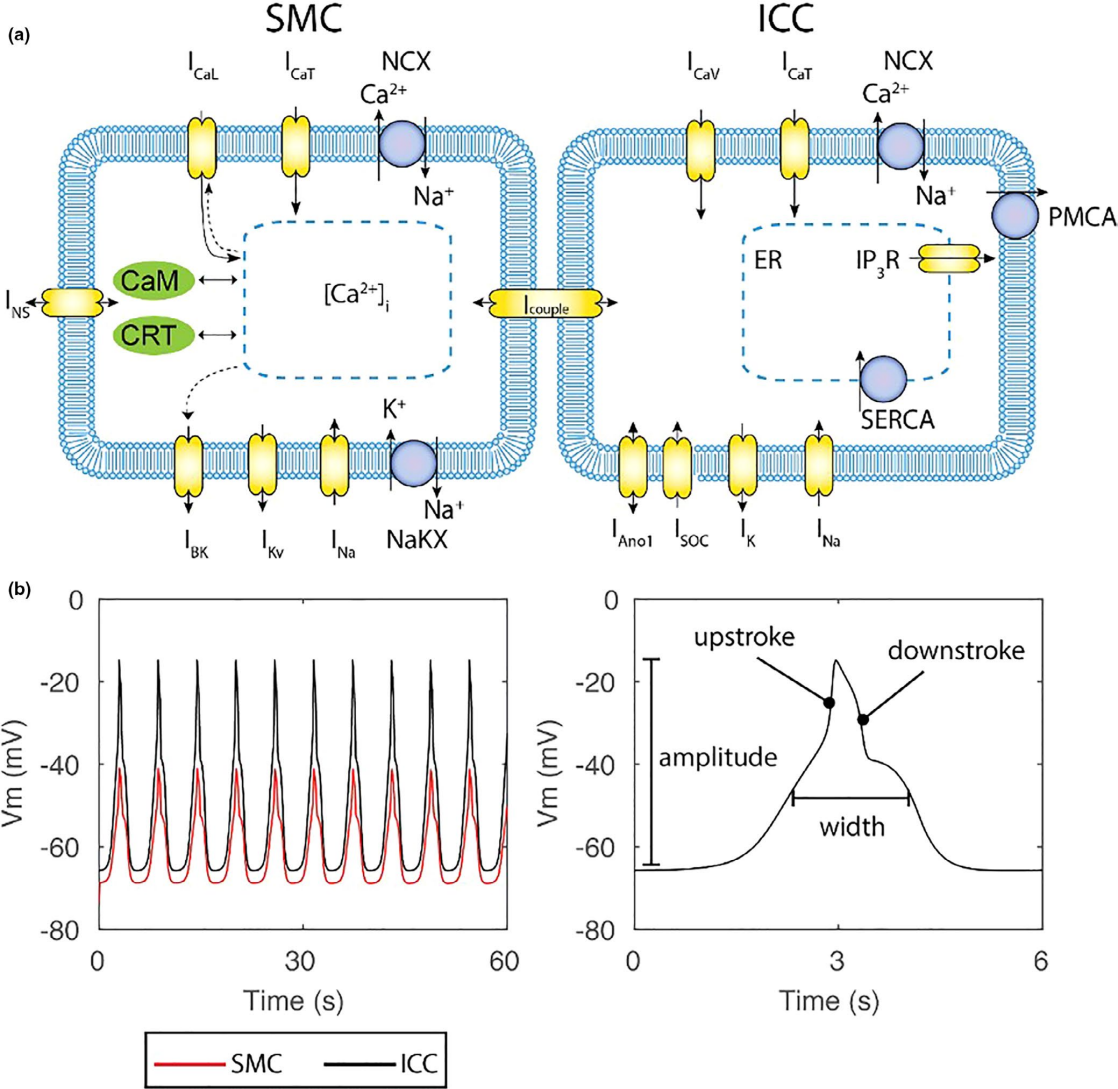
Mathematical model setup of SMC‐ICC jejunal slow waves. (a) A mathematical model of human jejunal SMC (Gizzi et al., 2010), was coupled to an ICC model (Lees‐Green et al., 2014) via a coupling conductance (Turkmani et al., 2019). Symbols of ion conductance (CaL, CaT, CaV, SOC, PMCA: calcium conductance; BK, Kv, K: potassium conductance; Na: sodium conductance; Ano1: chloride conductance; NS: non‐selective current; NCX, NaKX: exchangers; Icouple: coupling conductance) and intracellular components (CaM, CRT, ER, SERCA) used were identical to the original publications. A number of extracellular ion concentrations were changed to the mean baseline pre‐operative values. (b) Simulated baseline slow wave membrane potentials. The ICC generated slow waves (black) that successfully drove the slow waves in the SMC (red)

Biophysically based small intestine ICC and SMC mathematical models were applied to theoretically quantify the impact of extracellular electrolyte concentrations on cellular functions. The ICC model was based on a previously published intestinal slow‐wave model incorporating recent physiological data concerning Ano‐1 and all other ion channels active in ICC, developed by Lees‐Green et al. (2014). The SMC model was based on a human jejunal model developed by Poh, Corrias, et al. (2012). The two cell models were coupled via a gap junction model previously reported by Du et al. (2010). A schematic of the cell models and the main extracellular ion concentrations used to simulate the baseline intestinal slow waves is shown in Figure 1. These models have demonstrated predictive physiological reliability with regard to experimental and physiological variables in several studies (Du et al., 2009; Lees‐Green et al., 2014; Poh, Beyder, et al., 2012; Poh, Corrias, et al., 2012). The extracellular concentrations of NaCl were reduced up to 20% from the homeostatis state at 0.02% increments. The ODE15 s solver in MATLAB was used to solve both cell models with 1 × 10^−6^ for both relative and absolute tolerances with a maximum step of 1 ms. The simulations ran until steady‐state solutions were resolved.

**FIGURE 2:**
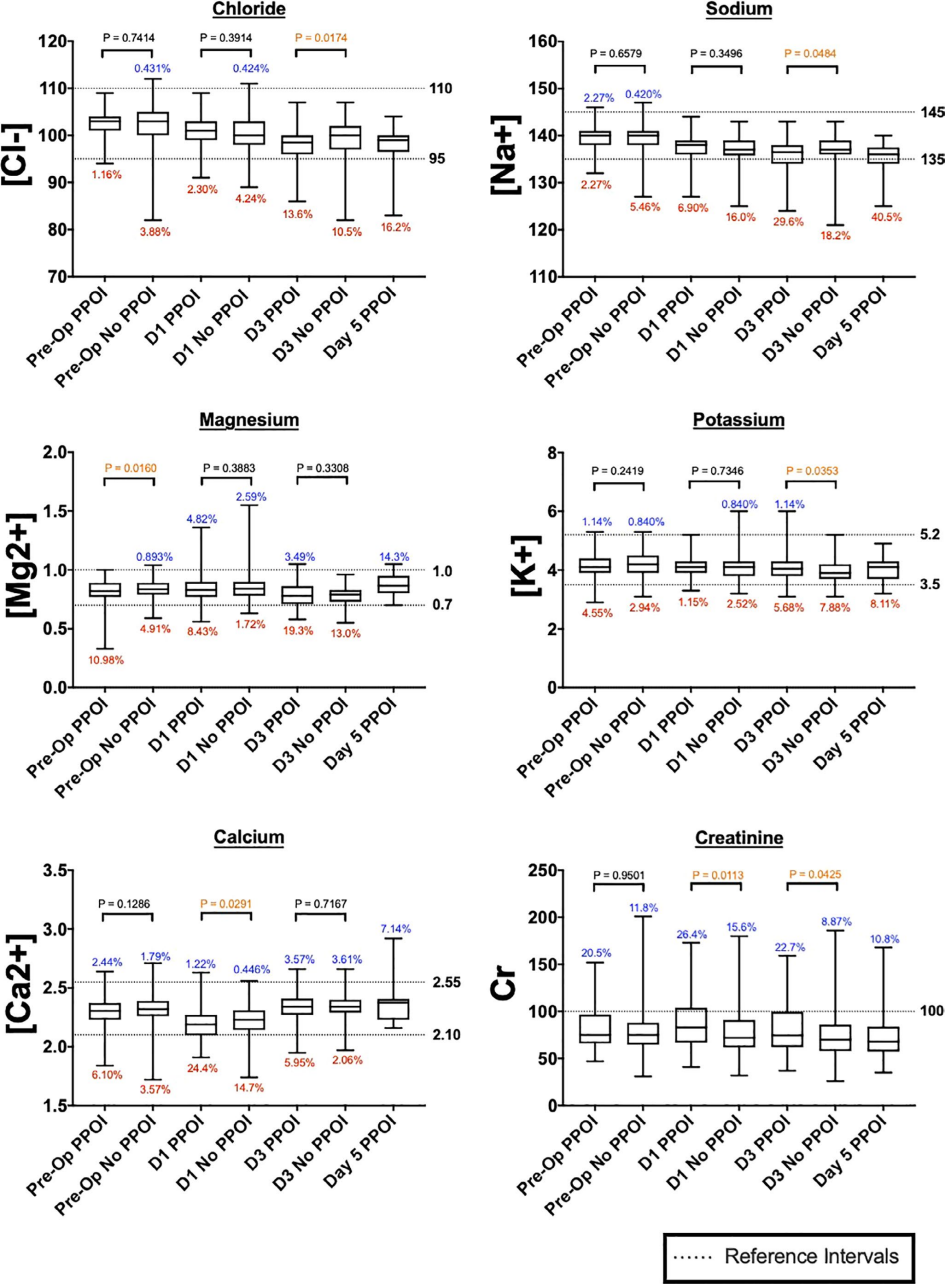
Box & Whisker Plot of Peri‐operative serum electrolyte concentrations/box & whisker plots for peri‐operative serum electrolyte trends for chloride, sodium, potassium, magnesium and calcium. All data points are displayed as mean with 95% confidence intervals

The clinical results (detailed below) were applied to inform in silico experimental physiological changes in the cellular models. Specifically, the extracellular concentrations of sodium and chloride were altered over time in accordance with the values observed experimentally in the PPOI group (Figure 2). The data were linearly interpolated on Days 2 and 3. The frequency, amplitude, upstroke, downstroke, and half‐amplitude width of the simulated slow‐wave membrane potentials were measured. The effects on calcium dynamics were investigated by plotting changes in intracellular calcium against membrane potentials at the different concentrations of sodium and chloride.

## 3 RESULTS

### 3.1 Clinical data

Data were analyzed from 327 patients, of which 88 developed PPOI according to the consensus definition applied (26.9%). Baseline characteristics are shown in Table 1. All serum reference ranges were taken from Labtests^®^ New Zealand, as appropriate for the laboratory electrolyte assays performed (Figure S1; Labtests).

**TABLE 1:** Baseline characteristics

	No PPOI (n = 239)	PPOI (n = 88)	All (n = 327)	*p*
Age (years)^a^	65.4	67.3	66.0	0.21
Sex (M:F)^b^	126:113	59:29	185:142	**0.021**
Race/ethnicity^b^				0.77
European	186	66	224	
Maori	8	5	13	
Pacific Islander	13	4	17	
Asian	32	13	45	
BMI (kg/m^2^)^a^	25.68	27.86	26.18	**0.005**
Indication^b^				0.36
Neoplasia	175	69	244	
IBD	23	7	30	
Diverticular disease	21	3	24	
Other	20	9	29	
Technique^b^				**0.002**
Open	117	60	177	
Laparoscopic‐assisted	71	12	83	
Laparoscopic	38	8	46	
Converted	12	8	20	
Procedure^b^				**0.0017**
Right hemicolectomy	34	19	53	
Anterior resection	83	28	111	
Abdominoperineal resection	10	4	14	
Total colectomy	5	7	12	
Reversal loop ileostomy	69	12	81	
Reversal Hartmann's or end‐ileostomy	17	14	31	
Other	21	4	25	

Note

Adapted from (Vather, Josephson, Jaung, Kahokehr, et al., 2015).

Abbreviations: F, female; IBD, inflammatory bowel disease; kg/m^2^, kilograms per meter squared; M, male.

Bold values indicate statistical significance (*P* < 0.05).

^a^ Nonparametric data expressed as median (IQR).

^b^ Chi‐squared contingency analysis.

POD1 calcium (2.19 vs. 2.22 L^−1^), POD3 chloride (98.0 vs. 99.2 mmol/L), and sodium (136.2 vs. 137.0 mmol/L) were lower in the PPOI group, whereas POD3 potassium was higher in the PPOI group (4.08 vs. 3.97 mmol/L; Figure 2; Table 2). When specifically analyzing deficits above or below the reference range, patients with PPOI were more likely to have an abnormality in POD1 sodium, magnesium, and calcium, and POD3 sodium (Table S1). POD3 sodium was outside the reference range in 29.6% of PPOI patients versus 18.2% in no PPOI patients (*p* = 0.026). POD3, chloride was outside the reference range in 13.6% of PPOI patients versus 10.5% in no PPOI patients, thus a statistically significant difference was not detected (*p* = 0.43).

**TABLE 2:** Peri‐operative electrolyte outcomes

	Chloride		Sodium		Potassium		Magnesium		Calcium	
	PPOI	No PPOI	PPOI	No PPOI	PPOI	No PPOI	PPOI	No PPOI	PPOI	No PPOI
Pre‐operative										
Number (*n*)	86	232	88	238	88	238	82	224	82	224
Mean (*SD*)	102.2 (3.1)	102.4 (3.8)	139.7 (2.7)	139.5 (2.9)	4.14 (0.44)	4.20 (0.42)	0.806 (0.12)	0.834 (0.08)	2.29 (0.13)	2.31 (0.12)
*p*	0.65	0.86	0.29	0.16	0.18					
Post‐operative day 1										
Number (*n*)	87	236	87	238	87	238	83	232	82	224
Mean (*SD*)	100.9 (3.3)	100.5 (3.5)	137.5 (2.7)	137.1(2.9)	4.12 (0.34)	4.11 (0.38)	0.837 (0.11)	0.848 (0.10)	2.19 (0.14)	2.22 (0.13)
*p*	0.34	0.43	0.76	0.39	**0.037**					
Post‐operative day 3										
Number (*n*)	88	200	88	203	88	203	86	193	84	194
Mean (*SD*)	98.0 (3.7)	99.2 (3.7)	136.2 (3.3)	137.0 (3.3)	4.08 (0.47)	3.97 (0.39)	0.790 (0.10)	0.779 (0.08)	2.34 (0.12)	2.34 (0.10)
*p*	**0.0082**	**0.026**	**0.035**	0.39	0.91					
Post‐operative day 5										
Number (*n*)	37	—	37	—	37	—	14	—	14	—
Mean (*SD*)	99	—	136	—	4.05	—	0.872	—	2.38	—

Abbreviations: IQR, interquartile range; *n*, number; PPOI, prolonged postoperative ileus; SD, standard deviation.

Bold values indicate statistical significance (*P* < 0.05).

**FIGURE 3:**
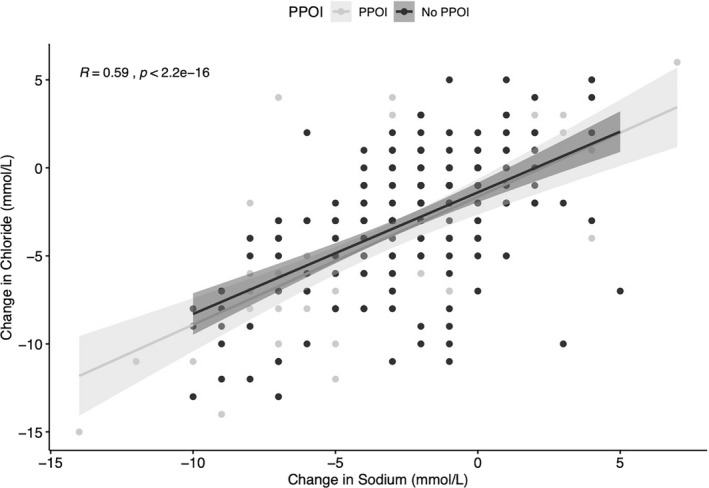
Sodium and chloride Pearson correlation plot/Pearson correlation plot, demonstrating a moderate to strong correlation with simultaneous changes in sodium and chloride from pre‐op to POD3, compared amongst the PPOI and no PPOI groups

The peri‐operative serum electrolyte trends are presented in Figure 2. Patients who developed PPOI were more likely to have a larger change in their median serum sodium and chloride concentrations from the pre‐operative setting to POD3 (mmol/L; PPOI = Δ−3.5 (+/−5) vs. no PPOI = Δ−2 (+/−4); *p* = 0.0047, and PPOI = Δ−4 (+/−5) vs. no PPOI = Δ−3 (+/−5); *p* = 0.011 for sodium and chloride, respectively; Table S2). Changes in sodium or chloride tended to occur together rather than in isolation, with 77.3% of patients (218/282) having a simultaneous decrease in sodium and chloride from the pre‐operative setting to POD3. A moderate positive correlation was observed for changes in sodium and chloride (*R* = 0.59); showing these trends often occurred synchronously (Figure 3). Of patients developing PPOI, 71% had a nasogastric tube placed as part of their management (34% prior to POD3). No patients without PPOI had a nasogastric tube placed.

Patients with PPOI had a longer length of stay, those with PPOI had 99% of the cohort stay >5 days compared to the no PPOI group who had 46% of the cohort stay >5 days. (Figure S2). Changes in serum sodium were also associated with a higher risk of minor and major complications as defined by Clavien‐Dindo grade (Figure S2). These complications did not include postoperative ileus.

### 3.2 Mathematical cell modeling

The baseline slow waves were simulated using the following mean extracellular ion concentrations based on the pre‐operative clinical data ([Cl−]_o_: 102.21 mmol/L; [Na+]_o_: 139.67 mmol/L; [K +]_o_: 4 mmol/L; [Ca2+]_o_: 2.29 mmol/L). The frequency of the simulated jejunal slow wave was 10.79 cpm, which was within range of normal human data based on in vivo electrophysiological evidence (Angeli et al., 2018). The simulated slow‐wave characteristics in ICC and SMC were: amplitude: 50.18 and 32.09 mV; width: 1.30 and 1.83 s; upstroke: 0.18 and 0.08 mV/ms; downstroke: −0.12 and −0.06 mV/ms.

**FIGURE 4:**
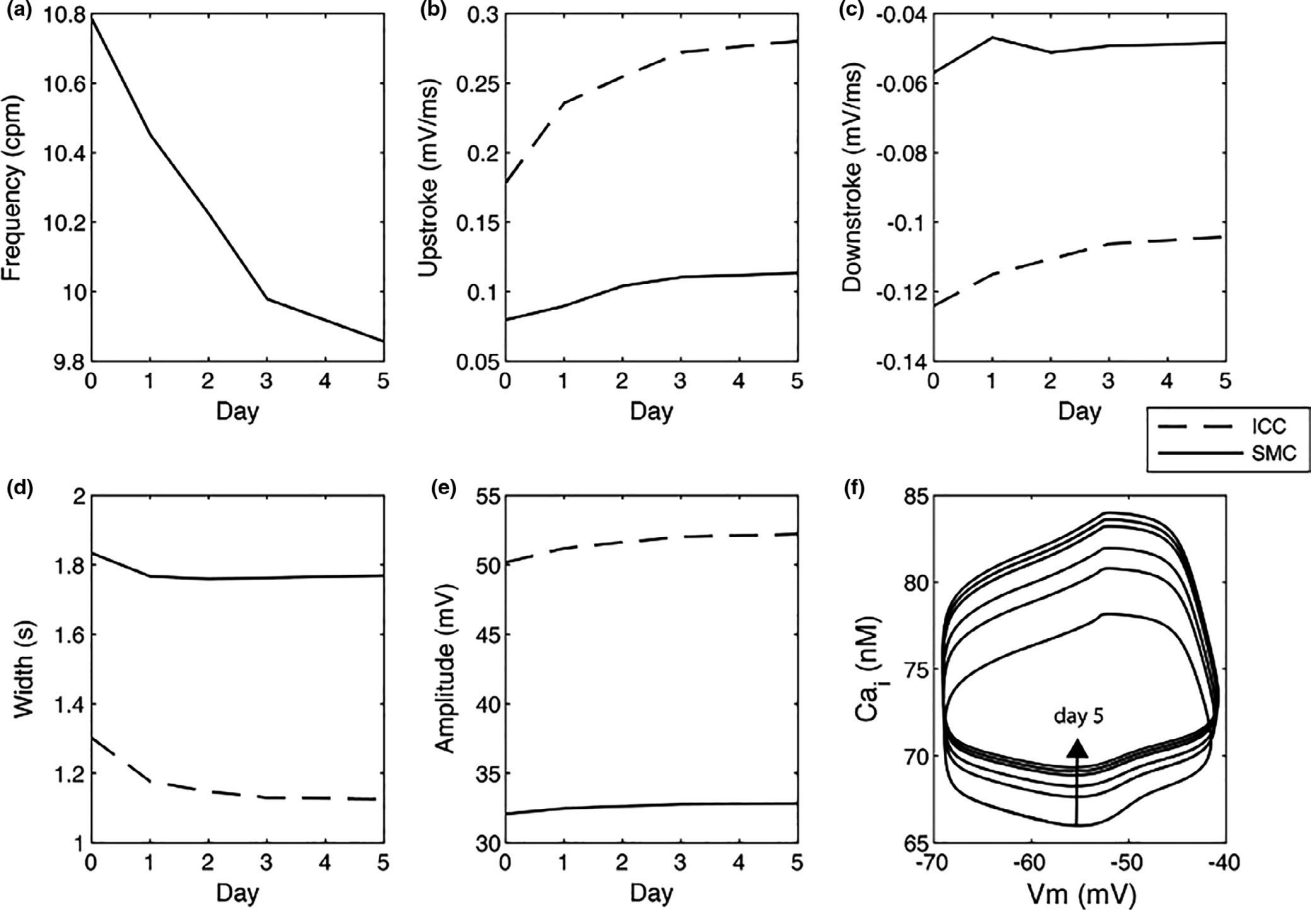
SMC and ICC slow‐wave characteristics/Theoretical analysis of changes in slow wave characteristics in response to change in [NaCl]o. (a) Frequency change. (b) Upstroke change. (c) Downstroke change. (d) Width change. (e) Amplitude change. (d) Calcium loops in SMC at different levels of [NaCl]o reductions

The clinical data above demonstrated that sodium and chloride declines tended to happen synchronously in patients with electrolyte abnormalities, and therefore, these changes were also modeled together in the in silico studies. Frequency was significantly impacted by equal reductions in [NaCl]_o_, in both ICC and SMC. In general, slow‐wave frequency reduced by up to 8.6% in compared to the baseline on Day 5 (Figure 4a).

The width of both ICC and SMC slow‐wave morphologies increased when [NaCl]_o_ was reduced. In general, the widths of slow waves were reduced by 175 and 65 ms on POD5 in ICC and SMC, respectively (Figure 4d). While the change in the width of slow waves in ICC due to reduction in [NaCl]_o_ was more pronounced compared to in SMC (13.5% vs. 3.5%). Amplitudes generally remained compared to the baseline values, increasing slightly as [NaCl]_o_ was reduced, by 2.03 mV (4%) and 0.74 mV (2.3%) in ICC and SMC, respectively (Figure 4e). There was no noticeable change in the resting membrane potentials of ICC and SMC, remaining at −66 and −73.5 mV, respectively. The changes in the upstrokes of slow waves due to the reductions in [NaCl]_o_ in ICC were less pronounced in ICC (18.8%) than in SMC (42%; Figure 4b). Moreover, the changes in the downstrokes of slow waves due to the reductions in [NaCl]_o_ in ICC (−16%) were comparable to SMC (−15%; Figure 4c).

A secondary effect of the reduction in [NaCl]_o_ was a predicted elevated accumulation of intracellular calcium in SMC (Figure 4d). The peak calcium cycle increased from 78.3 nM at the baseline to 84.2 nM on POD5, while the amplitude of calcium cycles also increased from 24.4 to 30.2 nM over the period.

## 4 DISCUSSION

This joint clinical and theoretical study has evaluated postoperative electrolyte derangements associated with ileus. It was found that abnormalities in POD1 calcium, and POD3 chloride, sodium, and potassium are associated with the occurrence of PPOI. Sodium and chloride concentrations trend downwards postoperatively, with patients developing PPOI having a more pronounced decline in these electrolyte levels. A biophysically based coupled ICC‐SMC mathematical model predicted a significant decline in jejunal slow‐wave frequency with reductions in sodium and chloride. Slow‐wave frequency was impacted more by a reduction in chloride concentration than sodium. Additionally, reductions in sodium and chloride resulted in several slow‐wave morphology changes, including increased slow‐wave duration and amplitude, exaggerated upstroke, and flattened down‐stroke.

This study reinforces that postoperative electrolytes may be a relevant factor in the pathophysiology ileus, as previously suggested (Miedema & Johnson, 2003). Notably, however, current management guidelines for postoperative GI recovery focus primarily on the monitoring and correction of sodium, and to some extent potassium (Mattei & Rombeau, 2006; Vather & Bissett, 2013), whereas chloride has been relatively overlooked. Chloride deserves focus given that Ano‐1 (a calcium‐activated chloride channel) is now understood to play a key role in slow‐wave pacemaker physiology, which is a key regulator of intestinal motility (Gomez‐Pinilla et al., 2009; Lees‐Green et al., 2014; Singh et al., 2014). Our results demonstrate that chloride may have a potential link to ileus, and moreover that changes in sodium and chloride routinely occur together in a manner that shows compounding implications for ICC and SMC function.

It is important to recognize the multifactorial nature and also the period of “obligatory” POI which is ubiquitous following major abdominal surgery (Vather et al., 2014). The data presented here do not imply that there is a primary causal relationship between electrolyte abnormalities and PPOI, as other factors such as neural and inflammatory are likely to be more significant promoters (Vather et al., 2014). In addition, this study cannot differentiate the degree to which the clinically documented electrolyte changes are contributory of ileus versus are non‐causal associations. Third‐space fluid shifts, IV fluid administration, dehydration, and the surgical stress response (including cytokine release and cortisol, ADH, and aldosterone secretion) are all factors known to potentially impact fluid and electrolyte balance in the peri‐operative period (Levitan & Mauer, 1968). For example, the relationship between sodium and chloride decline and ileus could potentially be indirectly explained by a heightened surgical stress response in these patients with greater concurrent ADH release. Decreased serum sodium concentrations are often attributed to increased water retention often due to ADH/Renin–angiotensin–aldosterone system (RAAS). Another confounding factor could be vomiting or nasogastric tube placement, which could contribute to a net loss of electrolytes and a decline in their concentration if patients rehydrate with hypotonic solutions. However, even if electrolyte abnormalities do not directly induce ileus, the present data do indicate that they could influence ileus severity or duration by altering contraction frequencies and slow‐wave electrophysiology and/or coupling.

The biophysically based models have previously shown predictive capability in real physiological scenarios and with experimental validation (Du et al., 2010; Lees‐Green et al., 2014; Poh, Corrias, et al., 2012). However, definitive interpretation is limited as the present cellular results are theoretical and are not yet directly validated in dedicated experimental studies. Overall, the changes in sodium and chloride resulted in decreased slow‐wave frequency and altered slow‐wave profile, that is, increased duration and amplitude with modified up‐/down‐stroke activity. Interestingly, however, the modeled calcium loops shift vertically with altered sodium and chloride levels, indicating more calcium released into and accumulation within the cytoplasm. It is currently unclear, however, whether this is an effective compensatory mechanism to increase contractile effort with greater calcium efflux as sodium and chloride decrease or, as merely a non‐homeostatic reactive response to sodium and chloride concentration changes. It is possible that the reduction in slow‐wave frequency could have disrupted motility more than the increase in intracellular calcium during individual slow‐wave cycles. The impact that the physiological changes predicted here may have on entrainment and calcium‐related effects now require further study.

A key functional metric of PPOI is the loss of motility, which should be linked to the calcium dynamics of the SMC model. Consideration should also be given to the heterogenous slow‐wave propagations in the GI tract (Lammers, 2015), and their role on the effects on motility. Previous investigations have developed detailed biomechanics models of the GI tract (Cherubini et al., 2017; Guarino et al., 2017; Pandolfi et al., 2017), as well as providing the link between electrophysiology and motility (Klemm et al., 2020). The detailed slow‐wave and calcium dynamic simulations results presented in this study could provide an update to guide future functional predictions of disease models due to PPOI.

Recent evidence has challenged the hypothesis of postoperative GI paralysis as a sine qua non of ileus (Huge et al., 2000; Vather et al., 2018; Wells et al., 2019). However, the precise abnormalities in motility contributing to both “obligatory” and “prolonged” ileus still remain poorly defined (Wells et al., 2019). Thus, a limitation of this clinical‐theoretical study is the inability to directly link electrolyte changes with specific motility patterns, which would be a useful goal for future work using experimental models.

In conclusion, this study documents and physiologically interrogates postoperative electrolyte derangements that are associated with ileus. Both sodium and chloride concentrations decrease in the days after major colorectal surgery, but by a greater margin in patients who develop PPOI, and with pronounced effects on gut electrophysiology predicted by biophysically based modeling. These electrolyte shifts may contribute to the impairment of gut motility observed in PPOI and to delayed recovery, but further research is needed to validate this finding and its clinical importance.

## CONFLICTS OF INTEREST

The other authors have no relevant conflicts of interest to declare.

## AUTHOR CONTRIBUTIONS

JAP: study design, data extraction, data analysis, primary drafting of the manuscript. All authors: study design and oversight, data interpretation, and critical review of the manuscript. All authors are qualified for authorship, have approved the final version of this manuscript, and agree to the accountability of its accuracy and integrity.

## Supporting information



Fig S1

Fig S2
