# Supplementary figures and images for: Relationships between serum electrolyte concentrations and ileus: A joint clinical and mathematical modeling study
Source: Physiol Rep. 2021 Feb 1;9(3):e14735. doi: 10.14814/phy2.14735 (PMC7851429; doi:10.14814/phy2.14735)

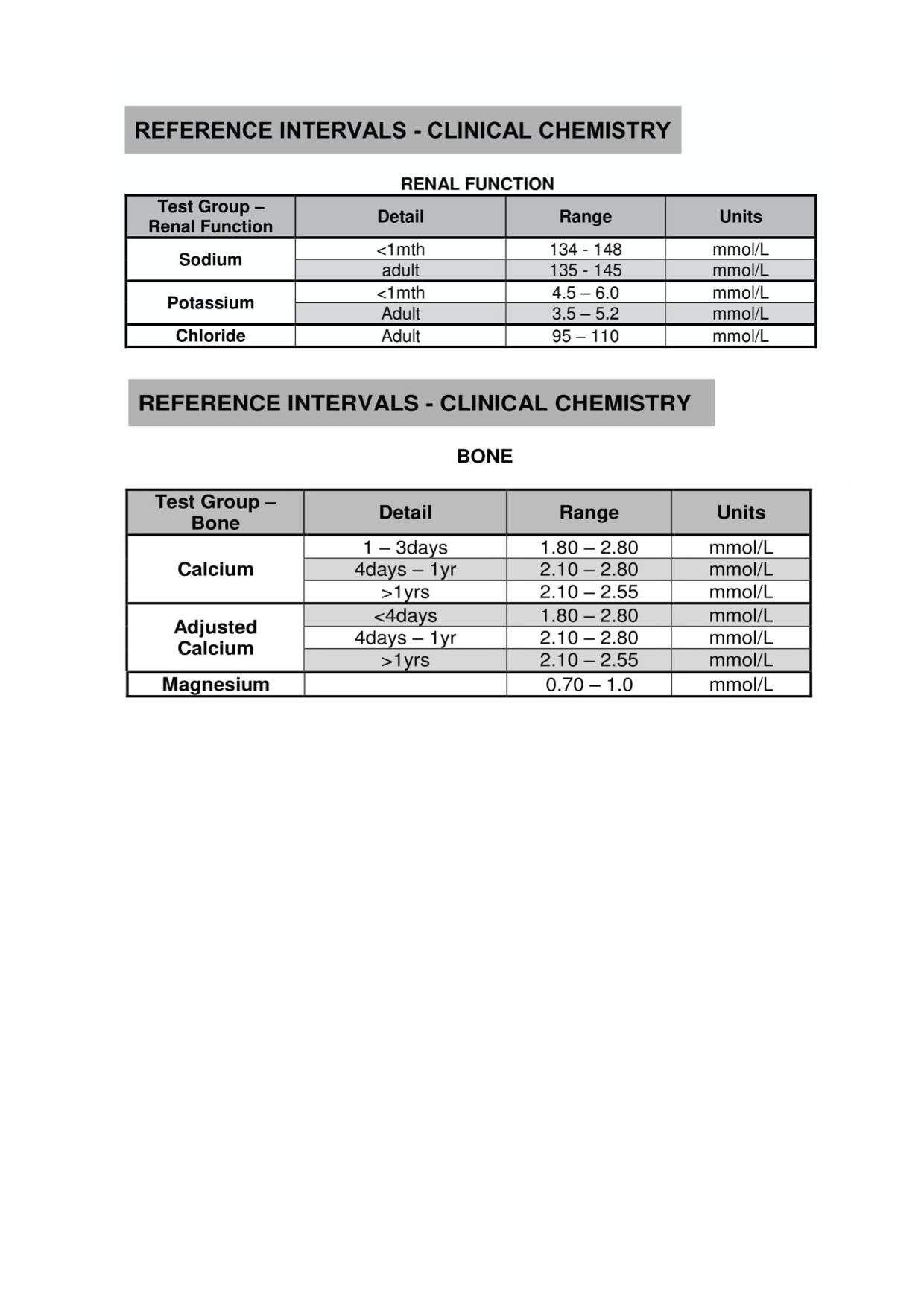

Supplement: Supplementary file 1 — Fig S1 [file PHY2-9-e14735-s001.jpeg]

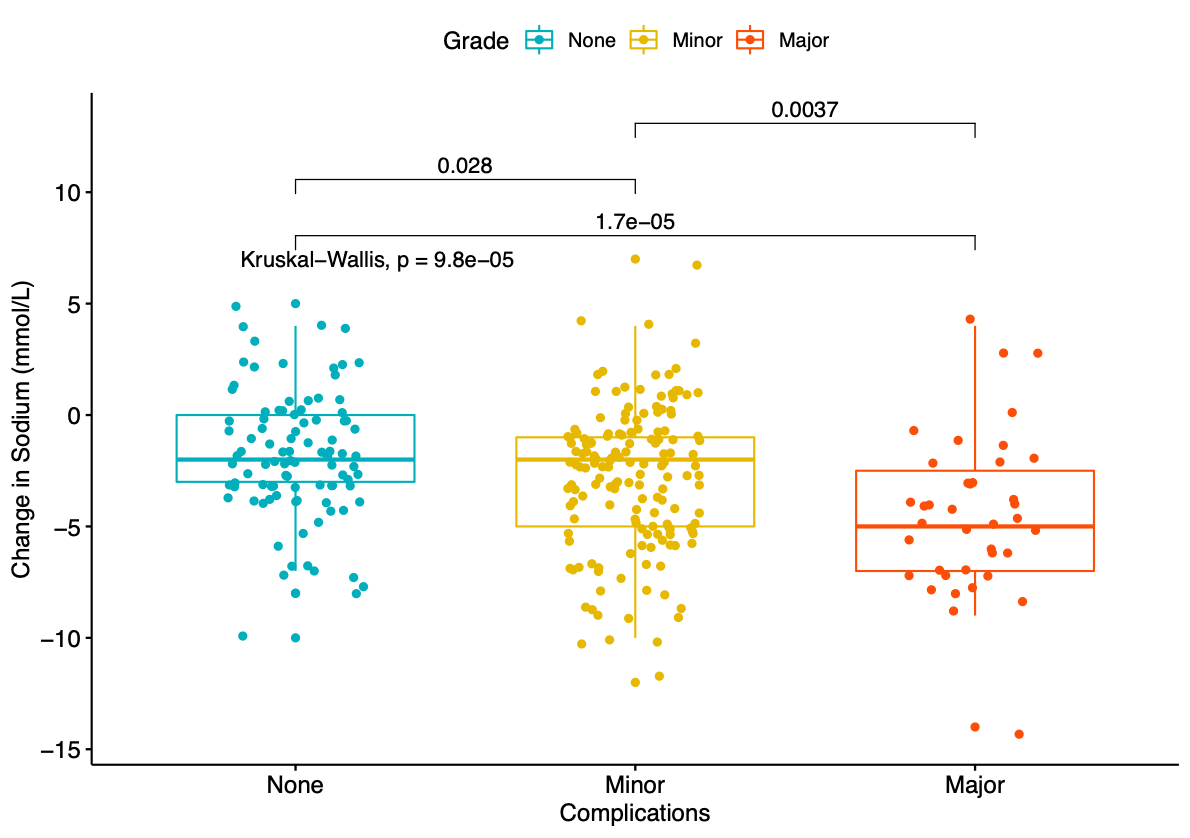

Supplement: Supplementary file 2 — Fig S2 [file PHY2-9-e14735-s002.jpeg]
